# Effects of metronidazole on colorectal cancer occurrence and colorectal cancer liver metastases by regulating *Fusobacterium nucleatum* in mice

**DOI:** 10.1002/iid3.1067

**Published:** 2023-11-28

**Authors:** Maijian Wang, Yong Li, Xuefeng Yang, Zhenxing Liu, Kai Wang, Dengmei Gong, Jida Li

**Affiliations:** ^1^ Department of General Surgery, Digestive Disease Hospital Affiliated Hospital of Zunyi Medical University Zunyi China; ^2^ Department of Oncology Guizhou Provincial People's Hospital Guiyang China; ^3^ Department of Pathology Affiliated Hospital of Zunyi Medical University Zunyi China; ^4^ Institute of Zoonoses, College of Public Health Zunyi Medical University Zunyi China

**Keywords:** colorectal cancer, colorectal cancer liver metastases, CT26 cells, DNA relative expression, *F. nucleatum*, gut microbiota, metronidazole

## Abstract

**Objective:**

Colorectal cancer (CRC) represents a leading cause of cancer‐related deaths. Metronidazole (MNZ) is exceedingly implicated in CRC. This study explored the roles of MNZ in mouse CRC occurrence and liver metastasis (CRLM).

**Methods:**

Male BALB/c nude mice were subjected to CRC and CRLM modeling, orally administration with MNZ (1 g/L) 1 week before modeling, and disease activity index (DAI) evaluation. Fresh stool and anal swab samples were collected on the morning of the 28th day after modeling. The relative expression of *Fusobacterium nucleatum* (*F. nucleatum*) DNA was assessed by quantitative polymerase chain reaction. After euthanasia, tumor tissues and liver tissues were separated and the tumor volume and weight change were measured. The liver tissues were stained with hematoxylin–eosin to quantitatively analyze the metastatic liver nodules. Malignant tumor biomarker Ki67 protein levels in liver tissues/DNA from stool samples were detected by immunohistochemistry/high‐throughput 16S rRNA gene sequencing. Bioinformatics analysis was performed on the raw sequence data to analyze microbial community richness (Chao1 index, ACE index) and microbial community diversity (Shannon index).

**Results:**

The DAI and *F. nucleatum* DNA relative expression in feces and anal swabs of the CRC and CRLM groups were raised and repressed after MNZ intervention. MNZ repressed tumor occurrence and growth in mice to a certain extent, alleviated CRLM malignant degree (reduced liver metastases and Ki67‐positive cell density/number), and suppressed CRC liver metastasis by regulating intestinal flora structure, which affected the intestinal characteristic flora of CRC and CRLM mice.

**Conclusion:**

MNZ suppressed CRC occurrence and CRLM in mice by regulating intestinal *F. nucleatum*.

## 1 INTRODUCTION

Colorectal cancer (CRC) accounts for approximately 10% of all of the annually diagnosed cancers and cancer‐related deaths all over the world, which is the third most prevalent cancer diagnosed in men and second most in women, and the incidence and mortality in women are about 25% lower than those in men.^1^ Inflammation is closely associated with tumors, with the systemic inflammatory response related to tumors considered as one of the significant indicators of tumor progression, and as a result, various serum systemic inflammatory markers can be used for tumor prediction and prognosis.^2^, ^3^ However, most CRC patients are diagnosed as advanced and have poor prognosis.^4^, ^5^ Moreover, in the late stage of CRC diagnosis, malignant hyperplasia and widespread metastasis have already occurred.^6^ The liver is the most common site of CRC metastasis, of which 15%–20% of the patients will be candidates for hepatectomy.^7^ Despite great advances in the diagnostic and therapeutic strategies, the prognosis still remains dismal because most of the patients develop metachronous or synchronous CRC liver metastases (CRLM).^8^

The mechanism of CRC occurrence and metastasis is identified as a multistep and multifactorial process, which may involve various genetic alterations and biological pathways.^9^ Studies have found that probiotics can be used as promising ingredients and play vital molecular roles for treating infectious diseases and intestinal‐related inflammation.^10^ In addition, prebiotics also play a crucial role in enhancing the intestinal microbiota, and can also be utilized as drugs to treat various infectious and inflammatory diseases relevant to the intestine (such as inflammatory bowel disease) and impede cancer progression.^11^ Besides, phytochemicals can influence the biological characteristics of CRC cells and perceptibly limit stem cell self‐renewal, migration, and invasion.^12^ The adaptive immune response at the tumor site is vital in the balance between tumor invasion and anticancer therapy.^13^ The gut microbiota colonizes the entire human gastrointestinal tract and co‐evolves through symbiotic relationships, affecting individual physiological, metabolic, nutritional, and immune functions, which directly participates in the protection of pathogen colonization by inducing direct killing, competing with nutrients, and enhancing the response of gut‐related immune banks.^14^ In CRC, the bacterial composition of the gut microbiota have many changes, indicating that ecological imbalance plays an important part in the occurrence of CRC.^15^ Many studies have also suggested that imbalance of gut microbiota can result in the occurrence of CRC.^16^, ^17^, ^18^ In all gut microbiota, *Fusobacterium nucleatum* (*F. nucleatum*), an anaerobic gram‐negative oral commensal bacterium, facilitates CRC by inducing Annexin A1.^19^ It promotes CRC metastasis by modulating KRT7‐AS/KRT7.^20^ Research suggests that *F. nucleatum* presence is related to the decreased T‐cell density in CRLM.^21^ Therefore, it is a new idea to reduce or delay CRC and its metastasis via repressing *F. nucleatum*. It has been reported that tumor microbiota vaccine targeting *F. nucleatum* have the latent capacity to ameliorate the prognosis of CRC.^22^ Fibroblast activating protein 2 (Fap2) is an essential membrane protein for *F. nucleatum* that can encourage bacterial adhesion to colon cells, recruit immune cells, and lead to tumor development, and designing a vaccine candidate containing Fap2 B‐ and T‐cell epitopes may serve as a promising therapeutic approach to intervene in *F. nucleatum*‐induced human CRC.^23^ Metronidazole (MNZ) is a pivotal drug for a variety of infections, which are induced by anaerobic bacteria, besides, MTZ reduced cell viability of DLD‐1 cell lines.^24^ *F. nucleatum* infections are routinely treated by MNZ.^25^ *F. nucleatum* induces tumor to resist to immunotherapy, while antibiotic MNZ treatment is able to reduce the abundance of *F. nucleatum*, making tumors re‐sensitive to immunotherapy in vivo.^26^ However, whether MNZ has an impact on CRC occurrence and CRLM by inhibiting *F. nucleatum* and regulating the related flora in mice remains largely unknown. This study investigated the effects of MNZ on CRC occurrence and CRLM through *F. nucleatum*, so as to provide a basis for revealing the internal relationship between *F. nucleatum* and its related flora, and CRC occurrence and CRLM, and provide a new idea for preventing CRC and its metastasis.

## 2 MATERIALS AND METHODS

### 2.1 Experimental animals

Male BALB/c nude mice (5–6 weeks old, weighing 18 ± 2 g) in the study were obtained from Vital River Laboratory Animal Technology (SCXK [Beijing] 2016‐0008). All mice were placed in a specific pathogen‐free room at 23 ± 1°C with 55% ± 5% humidity in 12 h light/dark cycles, and provided with sufficient food and water.^27^

### 2.2 Cell culture

Mouse CRC cell line CT26 was acquired from American Type Culture Collection (ATCC), which was incubated in the Dulbecco's modified Eagle medium (Gibco) containing 10% fetal bovine serum (Invitrogen) in an incubator comprising 5% CO_2_ at 37°C.

### 2.3 Establishment of CRC and CRLM models

CT26 cells in the logarithmic growth stage were collected, detached, dispersed into single‐cell suspension, and centrifuged at 1000 rpm for 5 min, and the supernatant was removed. The cell concentration was adjusted to 1 × 10^6^ cells/mL.^28^ Then, mice were injected with 0.2 mL CT26 cells into the posterior scapula or anorectum using a sterile syringe to establish CRC and CRLM models. After the injection, the mice were placed back into the feeding cages. The tumor volume on the 7th, 14th, 21st, and 28th days was measured and calculated as per the formula *V* (mm^3^) = [0.5 × *L* × *W* ^2^], where *L* and *W* represented length and width, respectively,^29^, ^30^ and the tumor was weighed on the 28th day. In addition, stool and anal swab samples were collected on the morning of the 28th day, and then the nude mice were euthanized by an intraperitoneal injection with excessive pentobarbital sodium (200 mg/kg) (Sigma‐Aldrich). Isolated tumor tissues or mouse liver tissues were employed for subsequent experimental analysis.

### 2.4 Animal treatment and grouping

A total of 30 mice were randomly allocated into the following five groups (six mice/group): Sham group (tail vein injection with 0.2 mL phosphate‐buffered saline [PBS]), CRC group (posterior scapula injection with 0.2 mL CT26 cell suspension), CRC + MNZ group (oral administration with 0.2 mL PBS solution with a MNZ concentration of 1 g/L 1 week before the establishment of CRC transplanted tumor models), CRLM group (anorectal injection with 0.2 mL CT26 cells), CRLM + MNZ group (oral administration with 0.2 mL PBS solution with a MNZ concentration of 1 g/L 1 week before the establishment of CRC liver metastasis models). The concentration of CT26 cells was 1 × 10^6^ cells/mL. MNZ was purchased from Sigma, with purity >98%, and the dose was determined based on previous studies.^31^, ^32^

### 2.5 Disease activity index assessment

After modeling, the weight changes, diarrhea, and bleeding of mice were observed every day, and the DAI was calculated as the sum of weight loss, diarrhea, and bleeding, with a weight loss score of 0 indicating no weight loss, 1 representing 1%–5% weight loss, 2 indicating 10% weight loss, 3 representing 10%–15% weight loss, and 4 indicating a weight loss over 15%. The benzidine test was conducted to assess whether there was blood in feces, which was scored from 0 to 4. The definition was as follows: 0 = blood; 2 points = positive blood occult; 4 points = severe bleeding. The severity of diarrhea was scored as 0–4 points, which was defined as follows: formed particles were 0 points; pasty and semiformed stools were 2 points; liquid stool was 4 points. The DAI values of each group were calculated on 1st, 3rd, 5th, 7th, 14th, 21st, and 28th days.

### 2.6 Stool sample collection and DNA extraction

Mouse fresh stool samples were collected on the morning of the 28th day. Pollution of sewage and microorganism was avoided during collection. The collected amount was >2 g. The samples were stored in the self‐zip bags and were repeatedly collected in three tubes. The self‐zip bags were placed into an ice box and immediately stored in a −80°C refrigerator. Anal swab samples were collected simultaneously. During collection, the anal circumference was cleaned with 70% alcohol. The special sterile swab was moistened by normal saline and inserted about 2–3 cm into the anus, gently rotated at the anal sphincter, stored in the sterile sampling solution, and immediately placed in a −80°C refrigerator. DNA in stool samples was extracted using the QIAamp DNA Stool Mini Kit (Nobleryder), and DNA in mucosal swab samples was extracted using the BIOG DNA Swab Kit (Biodai). The specific operation was carried out according to the instructions. The extracted DNA was detected using a NanoDrop spectrophotometer (Thermo Fisher Scientific). The A260/280 ratio of all samples was between 1.8 and 2.0. The extracted DNA was stored at −80°C.

### 2.7 Hematoxylin–eosin staining

According to the methods reported in previous research^33^ the liver tissues of nude mice were isolated, sectioned, dewaxed, and rehydrated. After washing with Milli‐Q water (Millipore), the tumor sections were stained with hematoxylin (H8070; Solarbio), stained with eosin (A600190; Sangon), placed in ethanol and xylene, and observed under a 200**×** optical microscope (Optical Instrument Factory) to quantitatively analyze the metastatic liver nodules.

### 2.8 Immunohistochemistry (IHC)

Based on the methods reported in previous research and with slight adjustments,^30^, ^34^ the tumor tissue sections were dewaxed and rehydrated in xylene (SINOPHARM) and gradient ethanol (SINOPHARM), immersed in distilled water, and treated with 3% H_2_O_2_ (SINOPHARM) to limit the activity of endogenous peroxidase. Then, the sections (4.5 μm) were incubated with anti‐Ki67 (HA721115; 1:500; HUAbio) at 37°C for 2 h. After rinsing with PBS buffer, the sections were incubated with secondary antibody goat anti‐rabbit HRP (SE134; 1:200; Solarbio) at room temperature for 1 h and then rinsed with PBS again. Subsequently, the sections were reacted with diaminobenzidine reagent (Amresco) for several seconds, counterstained with hematoxylin (Solarbio), dehydrated in gradient ethanol and xylene, fixed with glue, and imaged under an optical microscope. The number of Ki67‐positive cells in each section was quantitatively counted using the Image J software (version 1.8.0).

### 2.9 Reverse transcription quantitative polymerase chain reaction *(RT‐qPCR)*

Total RNA was extracted in one step using the TRIzol reagent (Invitrogen). The RNA concentration was determined using a UV spectrophotometer (Puyuan Spectral Instrument), and the integrity of RNA fragments was determined by gel electrophoresis. Reverse transcriptase M‐MLV (Aiyan Biotechnology) was applied for reverse transcription synthesis of cDNA. The specific operations were according to the kit instructions. The relative expression of DNA of *F. nucleatum* in feces and anal swabs was assessed by quantitative polymerase chain reaction (qPCR).^35^, ^36^ The PCR reaction conditions were as follows: a total of 40 cycles of denaturation at 95°C for 10 s, annealing at 60°C for 20 s, and extension at 72°C for 20 s. With β‐actin as the internal reference gene, the relative DNA expression of *F. nucleatum* as analyzed using the $2^{-∆∆C_{t}}$ method. The primers were synthesized by Sangon. Their sequences are shown in Table 1.

**Table 1:** Primer sequence.

Primer	Sequence
*F. nucleatum*	Forward: 5ʹ‐CTTAGGAATGAGACAGAGATG‐3′
	Reverse: 5′‐TGATGGTAACATACGAAAGG‐3′
β‐actin	Forward: 5′‐CCCAAGGCCAACCGCGAGAAGATG‐3′
	Reverse: 5′‐GTCCCGGCCAGCCAGGTCCAGA‐3′

### 2.10 16S rRNA gene sequencing

High throughput 16S rRNA gene sequencing was performed on the DNA samples of the samples. The v3–v4 region of the bacterial 16S rRNA gene was amplified by PCR using bacterial universal primers 338F 5ʹ‐ACTCCTACGGGAGGCAGCAG‐3′ and 806R 5′‐GGACTACHVGGGTWTCTAAT‐3′. The amplicons were extracted and purified by AxyPrep DNA gel extraction kit (Axygen Biosciences), and quantified using QuantiFluor™‐ST (Promega). The purified amplicons were sequenced on the Illumina MiSeq PE300 system (MajorBio).

### 2.11 Bioinformatics analysis

The original sequence data were screened through Barcode, primers, and other information to obtain high‐quality data. After optimizing, the sequences were compared using the SILVA database (https://www.arb-silva.de/). The sequences were clustered to generate operational taxonomic units (OTUs), and the generated OTU information was used to analyze the microbial community diversity and abundance index. The OTU with 97% similarity was used to analyze the microbial community richness (Chao1 index, ACE index) and microbial community diversity (Shannon index) unweighted Unifrac principal component analysis was employed to investigate the similarity between samples.

### 2.12 Statistical analysis

All data were processed by SPSS21.0 statistical software (IBM Corp. Armonk). Measurement data were expressed as mean ± standard deviation. One‐way repeated‐measures analysis of variance (ANOVA) or independent *t*‐test was used for comparisons between two groups and one‐way ANOVA was used for comparisons among multigroups, followed by Tukey's multiple comparisons test. *p* < .05 indicated statistical significance.

## 3 RESULTS

### 3.1 MNZ reduced the abundance of *F. nucleum* in CRC mice during liver metastasis

**Figure 1:**
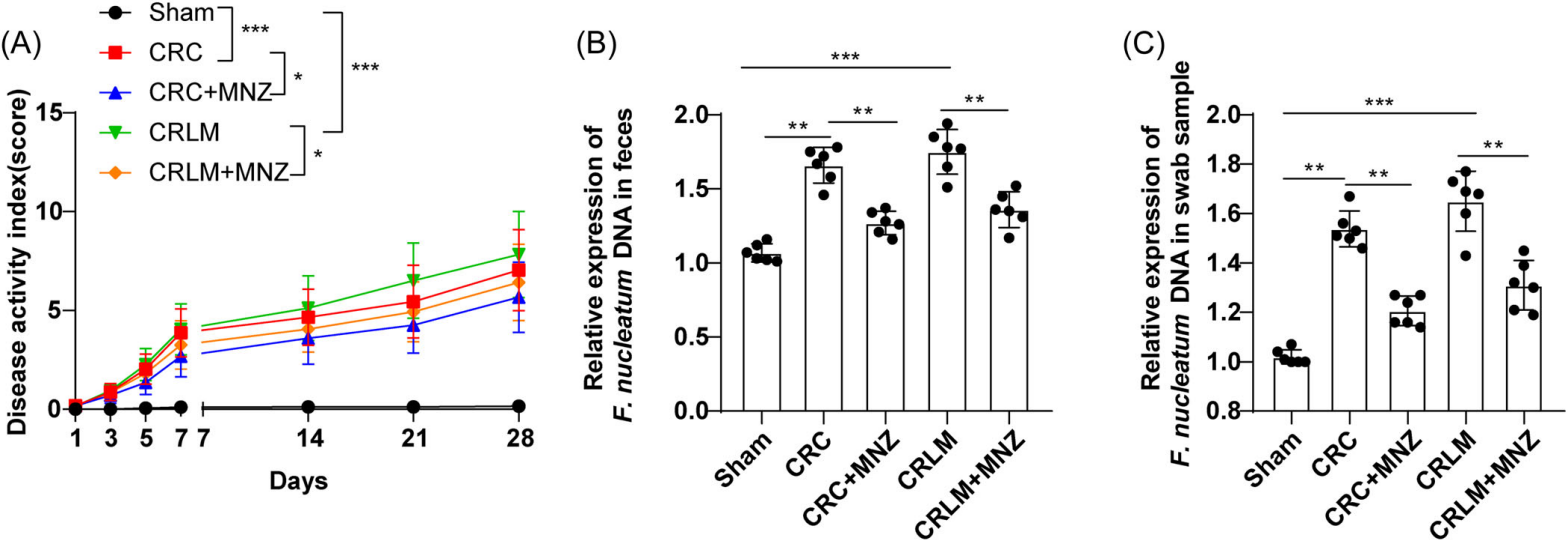
Effects of MNZ on the abundance of *F. nucleatum* during liver metastasis in CRC mice. (A) DAI of mice of each group was appraised. (B) The relative expression of *F. nucleatum* DNA in feces and anal swab samples was assessed by qPCR. *N* = 6. The data were expressed as mean ± standard deviation. One‐way ANOVA was adopted for data comparisons among multiple groups, followed by Tukey's multiple comparisons test. **p < .01, ***p < .001. CRC, colorectal cancer; DAI, disease activity index; MNZ, metronidazole; qPCR, quantitative polymerase chain reaction.

The mice were pretreated with 0.2 mL of PBS solution with a MNZ concentration of 1 g/L and injected with 0.2 mL of CT26 cells into the posterior scapula or anorectum using a sterile syringe 1 week later to establish CRC and CRLM models. First, the DAI of mice was estimated, which found that versus the Sham group, the DAI values of the CRC and CRLM groups were prominently elevated, while after MNZ intervention, the DAI values of both groups were notably diminished (all *p* < .05) (Figure 1A). The relative expression of *F. nucleatum* DNA in feces and anal swabs of each treatment group was assessed by qPCR. Compared with the Sham group, the relative expression of *F. nucleatum* DNA in feces and anal swabs of the CRC group and CRLM group was upregulated, while after MNZ intervention, the relative expression of *F. nucleatum* DNA in feces and anal swabs of CRC mice was decreased, and the relative expression of *F. nucleatum* DNA in the CRLM + MNZ group was lowered in contrast to the CRLM group (all *p* < .01) (Figure 1B,C). The results suggested that *F. nucleatum* was enriched in CRC mice, and MNZ reduced the abundance of *F. nucleatum* during liver metastasis in CRC mice.

### 3.2 MNZ inhibited tumor occurrence and growth in mice to a certain extent

**Figure 2:**
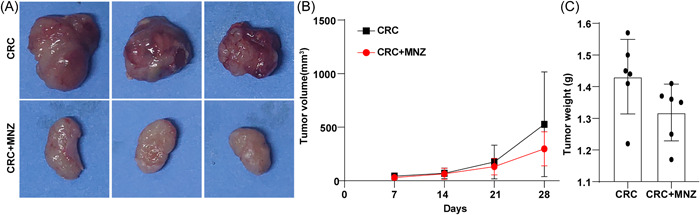
MNZ inhibited tumor occurrence and growth in mice to a certain extent. (A) Tumor body diagram of mice in each group; (B) The changes in tumor volume in nude mice were detected; (C) Tumor weight was measured. *N* = 6. The data were expressed as mean ± standard deviation. One‐way repeated measures ANOVA (B) or independent sample *t*‐test (C) were employed for data comparisons between two groups. MNZ, metronidazole.

The growth rate of xenotransplantation tumors was used to investigate the role of MNZ in CRC growth in vivo. The tumor volume was measured every 7 days, and the tumor was collected on the 28th day (Figure 2A). The tumorigenicity of CT26 cells in the CRC + MNZ group was lower than that in the CRC group, which was manifested as decreased tumor volume and weight, but there were no significant differences between the two groups (all *p* > .05) (Figure 2B,C). The results suggested that MNZ suppressed tumor occurrence and growth in mice to some extent.

### 3.3 MNZ alleviated the malignant degree of CRC liver metastasis

**Figure 3:**
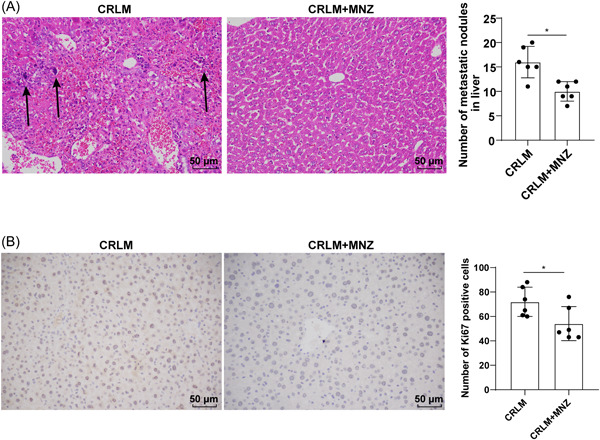
MNZ alleviated the malignant degree of CRC liver metastasis. (A) The liver tissues were stained with H&E and the liver metastatic nodules were quantitatively analyzed, and metastatic nodules were indicated by black arrows; (B) The expression of Ki67‐positive cells in liver tissues was assessed by IHC. *N* = 6. The data were expressed as mean ± standard deviation. Independent sample *t*‐test was employed for data comparison between two groups. *p < .05. CRC, colorectal cancer; H&E, hematoxylin–eosin; IHC, immunohistochemistry; MNZ, metronidazole.

We further investigated the effects of MNZ on CRLM malignancy. The results of hematoxylin–eosin (H&E) staining revealed that compared with the CRLM + MNZ group, the CRLM group cells in hepatic tissue were irregular, with swollen and abnormal nuclei and more chromatin, while after MNZ treatment, tumor cell density was decreased and irregular cells were reduced. Moreover, compared with the CRLM group, the number of liver metastatic nodules in the CRLM + MNZ group was decreased (*p* < .05) (Figure 3A).

Ki67 protein in liver tissues was detected and quantified by immunohistochemistry. Ki67‐positive cell density and number in the CRLM + MNZ group were diminished (*p* < .05) (Figure 3B). These results suggested that MNZ alleviated CRLM malignant degree.

### 3.4 MNZ repressed CRC liver metastasis by regulating intestinal flora structure in mice

**Figure 4:**
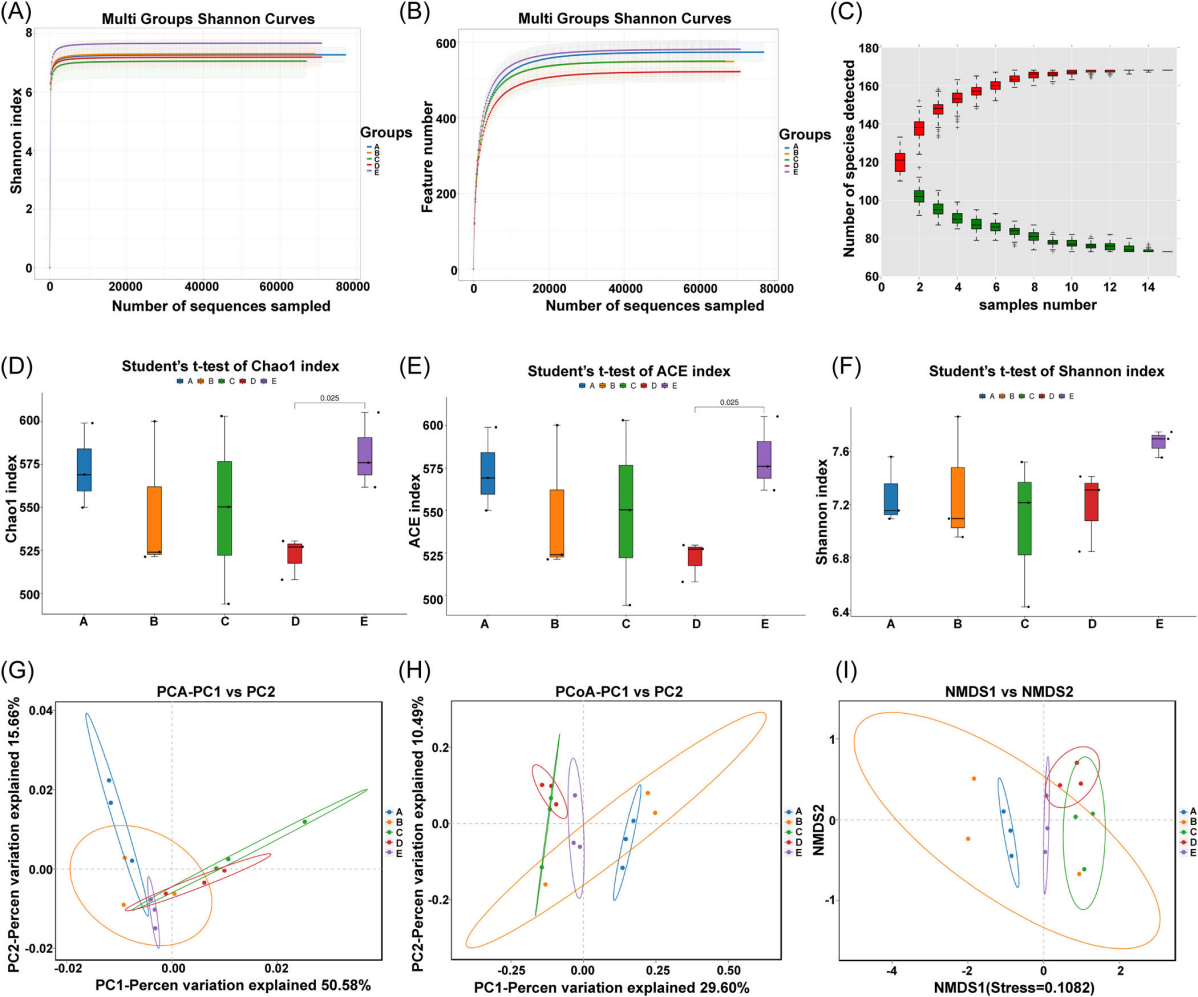
MNZ repressed CRC liver metastasis by regulating intestinal flora structure in mice. (A) Shannon index curve; (B) sample dilution curve; (C) species accumulation curve at genus level; (D) Chao1 index; (E) ACE index; (F) Shannon index; (G) principal component analysis (PCA): each point represented a sample, and different colors represented different samples/groups. The abscissa represented the first principal component, the percentage represented the contribution value of the first principal component to the sample difference, the ordinate represented the second principal component, and the percentage represented the contribution value of the second principal component to the sample difference. The two samples were closer when the composition of the two samples were more similar; (H) principal coordinates analysis (PCoA) showed that the samples were closer on the coordinate map when they had a greater similarity; (I) for nonmetric multidimensional scaling (NMDS) analysis, the points in the figure represented each sample, different colors represented different groups, and the distance between points represented the degree of difference. When the stress was less than 0.2, it indicated certain reliability of the NMDS analysis. The samples were closer in the coordinate map when they had the higher similarity. (A) represented the Sham group, (B) represented the CRC group, (C) represented the CRC + MNZ group, (D) represented the CRLM group, and (E) represented the CRLM + MNZ group. CRC, colorectal cancer; CRLM, CRC liver metastases; MNZ, metronidazole.

Alpha diversity assesses the richness and diversity of gut microbiota.^36^ Based on the OTU results, the alpha diversity index of each group at the OTU similarity level (97%) was calculated, including ACE index and Chao1 index (representing abundance) and Shannon index (representing microbial diversity). A higher ACE or Chao1 index represented a higher richness of the flora. A higher Shannon represented a higher diversity of the flora. The microbial diversity and abundance of the CRC group were decreased (Figure 4A–F). Quantitative analysis manifested that compared with the Sham group, the Chao index and ACE index of the CRC group and the CRLM group were diminished, indicating that the flora abundance of CRC mice was lower than that of Sham mice. In addition, the Shannon index of the CRC group was lowered than that of the Sham group, indicating a decreased microbial diversity in mice. After MNZ intervention, the richness and diversity of the flora in the CRC and CRLM groups were both elevated (all *p* < .05) (Figure 4D–F). In addition, β‐diversity analysis demonstrated that all five groups of intestinal microflora had obvious clustering (Figure 4G–I). The microflora structure of the CRC group, CRLM group, and Sham group was quite different. After MNZ intervention, the microflora structure significantly changed.

### 3.5 Effect of MNZ on characteristic intestinal microflora of CRC and CRLM mice

**Figure 5:**
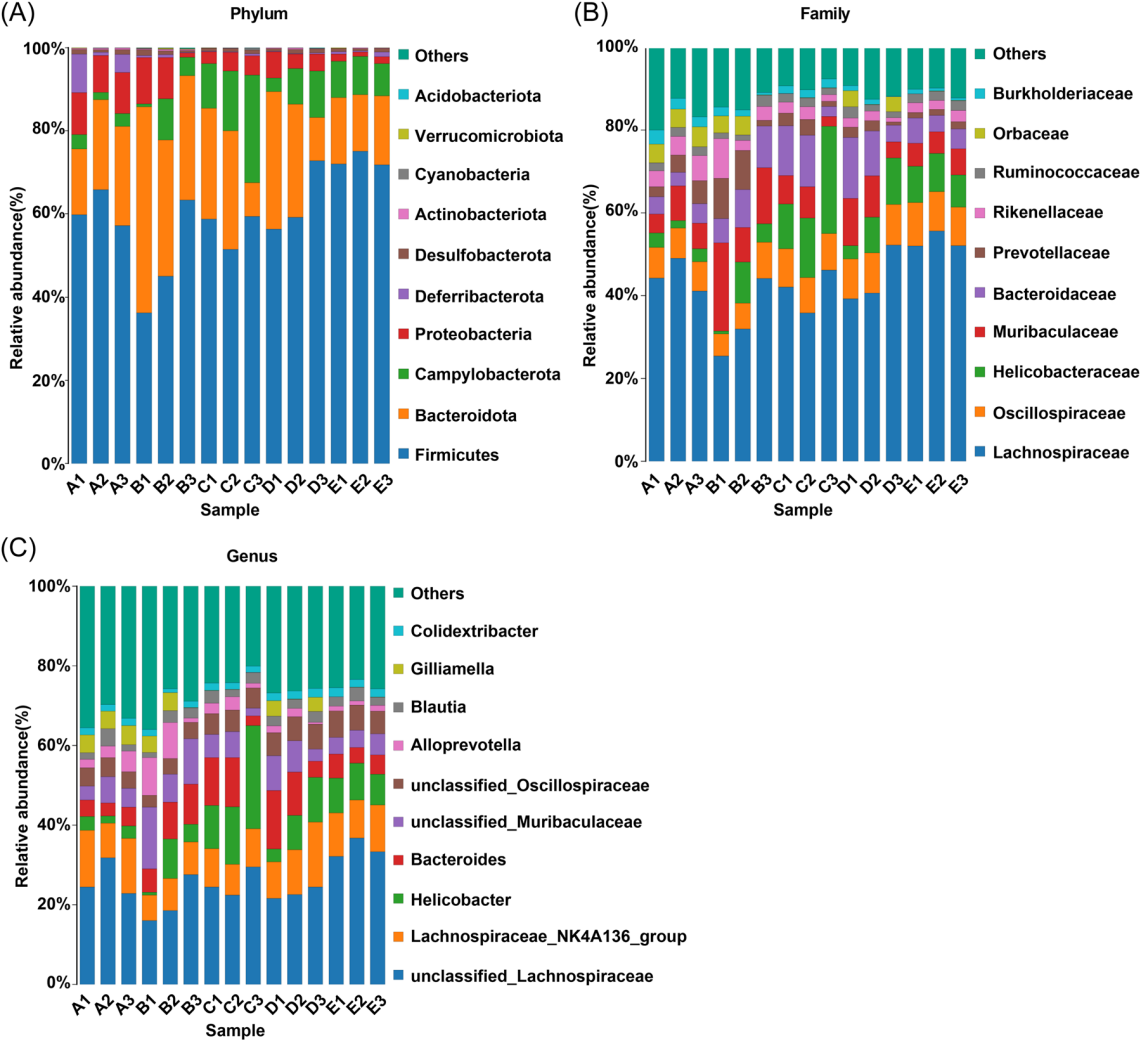
Effect of MNZ on characteristic intestinal microflora of CRC and CRLM mice. Relative abundance changes of intestinal characteristic flora of mice in each group at the level of (A) phylum; (B) family; (C) genus. The abscissa was the sample name (A–E represented the Sham group, CRC group, CRC + MNZ group, CRLM group, and CRLM + MNZ group, respectively); the ordinate was the relative abundance percentage. Different colors represented different species; the stacking column was a taxon with relative abundance of top 10 at each taxonomic level. MNZ, metronidazole; CRC, colorectal cancer; CRLM, CRC liver metastases.

Next, we investigated the differences of major microflora in each group at the taxonomic level. The results of phylum, family, and genus levels were used to analyze the composition of intestinal microbiota in different groups of mice. At the phyla level, the first five species of bacteria in each treatment group were *Firmicutes*, *Bacteroidota*, *Campylobacterota*, *Proteobacteria*, and *Deferribacterota*. Among them, *Firmicutes* and *Bacteroidota* were the main dominant flora, accounting for the largest proportion in intestinal flora. The contents of *Firmicutes*, *Proteobacteria*, and *Deferribacterota* in the CRC group were lower than those in the Sham group, while the contents of *Bacteroidota* and *Campylobacterota* were increased. After MNZ intervention, the content of *Firmicutes* in the CRC group and CRLM group was augmented and the content of *Bacteroidota* was reduced (Figure 5A).

At the family level, the first five species of bacteria in each group were *Lachnospiraceae, Oscillospiraceae, Helicobacteraceae, Muribaculaceae*, and *Bacteroidaceae*. Compared with the Sham group, *Lachnospiraceae* was diminished in the CRC group, while *Muribaculaceae* was facilitated. MNZ intervention increased the content of *Lachnospiraceae* and decreased the content of *Muribaculaceae* in CRC mice, and maintained the *Lachnospiraceae* content in CRLM mice. There was no significant difference in the content of *Oscillospiraceae* in each group (Figure 5B).

At the genus level, the dominant flora with relatively higher abundance were *unclassified Lachnospiraceae, Lachnospiraceae NK4A136 group*, *Helicobacter, Bacteroides*, and *unclassified Muribaculaceae*. The contents of *unclassified Lachnospiraceae* and *Lachnospiraceae NK4A136 group* in the CRC group were lower than those in the Sham group, while the contents of other three types of bacteria were increased. After MNZ intervention, the content of *unclassified Lachnospiraceae* in the CRC group and CRLM group was upregulated, which was more similar to those in Sham mice (Figure 5C).

## 4 DISCUSSION

CRC is the third most prevalent cancer and the fourth most prevalent cause of cancer‐related death all over the world, accounting for about 1.2 million new cases and 600,000 deaths annually, with its incidence low at ages younger than 50 years, but strongly increasing with age.^37^ Extrinsic and intrinsic inflammations can both lead to immunosuppression, thus offering an optimal background for tumor development.^38^ MNZ treatment rapidly reduces genital inflammation through its effects on bacterial vaginosis‐associated bacteria.^39^ This study found that MNZ suppressed the occurrence of CRC and CRLM by inhibiting *F. nucleatum*.

*F. nucleatum* infections are routinely treated by MNZ.^25^ MNZ represses the cell viability of DLD‐1 CRC cell lines.^24^ Some therapies, such as probiotics, diets, prebiotics, stool microbiota transplantation, ursodeoxycholic acid, and engineered bacteria, can mitigate inflammatory bowel diseases by means of bile acids and gut microbiota restoration.^40^ Our results revealed that the relative DNA expression of *F. nucleatum* was facilitated in anal swabs and feces of CRC mice and CRLM mice, but diminished after MNZ treatment. In addition, MNZ blocked the occurrence and growth of tumors in mice to some extent. Consistently, the feces samples and tumor microenvironment of patients with CRC are enriched by *F. nucleatum*.^41^ Treatment of mice bearing a CRC xenograft with the antibiotic MNZ suppressed *F. nucleatum* load, overall tumor growth, and cancer cell proliferation.^42^ In brief, MNZ suppressed the abundance of *F. nucleatum* during CRLM in mice and blocked the growth of CRC.

Among all organs, liver metastasis is the most prevailing, which makes the treatment of CRC challenging.^43^ To further study the role of MNZ in the malignancy of CRLM, we treated the mice with MNZ and established CRLM models and discovered that after MNZ treatment, CRLM mice manifested decreased hepatic metastatic nodules, reduced density of tumor cells, and less irregular cells. Ki67 is a kind of proliferation marker, which can indicate the growth of metastatic liver tumors.^34^ Our results demonstrated that the density and number of Ki67‐positive cells in CRLM mice after MNZ treatment were reduced. However, there is no report on the effect of MNZ on the malignant degree of CRLM. This study discovered that MNZ alleviated the malignant degree of CRLM for the first time.

Dysbiosis in the gut microbiota composition due to genetic or environmental variations can disrupt the immune system and promote a variety of diseases including CRC.^16^ Intestinal microbiota diversity and composition gradually returned to healthy control levels after the application of eradication therapy containing MNZ.^44^ Our results discovered that after MNZ intervention, the richness and diversity of flora in the CRC and CRLM mice were raised, and the microflora structure was significantly changed. Functional studies in animal models pinpoint the properties of a variety of bacteria in CRC occurrence, such as *F. nucleatum* and certain strains of *Escherichia coli* and *Bacteroides fragilis*.^45^ Studies have identified a correlation between gut microbiota composition and gastrointestinal cancers.^46^ In addition, at the Phylum level, *Bacteroida* and *Firmicutes* were the most dominant. After MNZ intervention, the content of *Firmicutes* in the CRC and CRLM groups was increased and *Bacteroidota* was decreased. At the Family level, MNZ intervention augmented the content of *Lachnospiraceae* and reduced *Muribaculaceae* in CRC mice. At the Genus level, after MNZ intervention, the unclassified *Lachnospiraceae* content in the CRC and CRLM groups was upregulated, closer to the Sham mice. A disturbance of the fragile balance between different bacteria species in human gut towards upregulated levels of bacteria *Bacteroides fragilis* and *F. nucleatum* is related to an elevated risk of CRC.^47^ A previous study has also elicited an abundance of *Firmicutes* but a decrease in *Bacteroides* in CRC mice.^48^ The downward trend in the abundance of *Firmicutes, Planctomycetes*, and *Tenericutes* occurred in horses due to MNZ administration.^49^ MNZ affects the course of inflammatory bowel disease by reducing bacteria concentrations in the gut lumen and altering intestinal microbiota composition.^50^ MNZ has been used to reduce gastrointestinal symptoms in children susceptible to *Dientamoeba Fragilis* and MNZ treatment has short‐term impacts on the abundance of some bacterial genera.^51^ The use of a specific antibiotic MNZ eliminates the adverse effects of oral microbiome fluctuations on CRC radiotherapy.^52^ Despite the vital role of MNZ, it is well tolerated and has mild to moderate side effects such as abdominal pain, nausea, and diarrhea.^53^ Besides, long‐term MNZ treatment will trigger oxidative stress and thiamine deficiency.^54^ MNZ had effects on characteristic intestinal microflora of CRC and CRLM mice. To conclude, MNZ suppressed CRC liver metastasis by modulating the structural composition and abundance of intestinal flora in mice.

In summary, this study supported that MNZ suppressed the occurrence of CRC and CRLM by regulating *F. nucleatum*. This study combined basic science with clinical practice, strived to develop new treatment methods to prevent CRC and CRLM while solving basic science problems, and provided theoretical and experimental bases for the follow‐up treatment strategy of “targeting gut microbiota,” which was a preliminary exploration in this field.

## STUDY LIMITATION

However, the study period was short, and as a result, it was difficult to evaluate the long‐term effects of MNZ. In addition, the sample size was relatively small. The specific mechanism of MNZ regulating the occurrence of CRC and CRLM was not thoroughly studied. Genes play a leading part as prognostic biomarkers in cancer disease detection.^55^ However, due to our lack of experience in bioinformatics analysis and time cycles, this study is currently unable to analyze the differential expression of CRC and CRLM‐related genes. If conditions permit, we will carry out multicenter and large sample research with extended research time. Moreover, further work is needed to explore the related signal pathways of MNZ inhibiting the growth of *F. nucleatum*, explore the mechanism of MNZ regulating CRC and CRLM, and analyze the differential expression of CRC and CRLM‐related genes through utilizing bioinformatics analysis software.

## AUTHOR CONTRIBUTIONS

Maijian Wang, Yong Li, Xuefeng Yang, and Zhenxing Liu performed the research. Maijian Wang, Yong Li and Jida Li designed the research study. Yong Li and Jida Li contributed essential reagents or tools. Maijian Wang, Dengmei Gong, and Zhenxing Liu analyzed the data. Maijian Wang wrote the paper. All authors have read and approved the final manuscript.

Maijian Wang and Jida Li are the guarantor of integrity of the entire study and contributed to the manuscript review; Xuefeng Yang, Zhenxing Liu contributed to the study concepts; Maijian Wang, Dengmei Gong contributed to the study design; Xuefeng Yang, Dengmei Gong contributed to the definition of intellectual content, manuscript preparation; Jida Li, Xuefeng Yang contributed to the literature research; Yong Li, Zhenxing Liu contributed to the experimental studies; Jida Li, Yong Li contributed to the data acquisition; Maijian Wang, Zhenxing Liu contributed to the data analysis; Jida Li, Yong Li, and Kai Wang contributed to the statistical analysis; Maijian Wang, Kai Wang contributed to the manuscript editing.

## CONFLICT OF INTEREST STATEMENT

The authors declare no conflicts of interest.

## ETHICS STATEMENT

The study was authorized by the academic ethics committee of Affiliated Hospital of Zunyi Medical University (Approval number: KLL‐2019‐004). All procedures were strictly implemented under the Guide for the Care and Use of Laboratory Animals. All laboratory procedures were used to minimize the pain of mice.

## Data Availability

The data that support the findings of this study are available from the corresponding author upon reasonable request.

## References

[1] Dekker E , Tanis PJ , Vleugels JLA , Kasi PM , Wallace MB . Colorectal cancer. Lancet. 2019;394:1467‐1480. 31631858 10.1016/S0140-6736(19)32319-0

[2] Yamamoto T , Kawada K , Obama K . Inflammation‐related biomarkers for the prediction of prognosis in colorectal cancer patients. Int J Mol Sci. 2021;22:8002. 34360768 10.3390/ijms22158002 PMC8348168

[3] Chen JH , Zhai ET , Yuan YJ , et al. Systemic immune‐inflammation index for predicting prognosis of colorectal cancer. World J Gastroenterol. 2017;23:6261‐6272. 28974892 10.3748/wjg.v23.i34.6261 PMC5603492

[4] Schmidhammer H , Smith CFC , Erlach D , et al. Synthesis and biological evaluation of 14‐alkoxymorphinans. 3. Extensive study on cyprodime‐related compounds. J Med Chem. 1990;33:1200‐1206. 2157011 10.1021/jm00166a018

[5] Fan A , Zhao X , Liu H , et al. eEF1A1 promotes colorectal cancer progression and predicts poor prognosis of patients. Cancer Med. 2023;12:513‐524. 35607944 10.1002/cam4.4848 PMC9844609

[6] Walsh JME , Terdiman JP . Colorectal cancer screening: scientific review. JAMA. 2003;289:1288‐1296. 12633191 10.1001/jama.289.10.1288

[7] Akgül Ö . Role of surgery in colorectal cancer liver metastases. World J Gastroenterol. 2014;20:6113‐6122. 24876733 10.3748/wjg.v20.i20.6113 PMC4033450

[8] Martinou E , Moller‐Levet C , Karamanis D , Bagwan I , Angelidi AM . HOXB9 overexpression promotes colorectal cancer progression and is associated with worse survival in liver resection patients for colorectal liver metastases. Int J Mol Sci. 2022;23:2281. 35216396 10.3390/ijms23042281 PMC8879839

[9] Hou P , Meng S , Li M , et al. LINC00460/DHX9/IGF2BP2 complex promotes colorectal cancer proliferation and metastasis by mediating HMGA1 mRNA stability depending on m6A modification. J Exp Clin Cancer Res. 2021;40:52. 33526059 10.1186/s13046-021-01857-2 PMC7851923

[10] Das NC , Patra R , Dey A , Mukherjee S . Prebiotics, Probiotics and Nutraceuticals. Probiotics as efficacious therapeutic option for treating gut‐related diseases: molecular and immunobiological perspectives, 2022:69‐93.

[11] Patra R , Mitra S , Das NC , Mukherjee S . Probiotics and Nutraceuticals. Probiotics as efficacious therapeutic option for treating gut‐related diseases: molecular and immunobiological perspectives, 2022:133‐154.

[12] Liao W , Zhang L , Chen X , et al. Targeting cancer stem cells and signalling pathways through phytochemicals: a promising approach against colorectal cancer. Phytomedicine. 2023;108:154524. 36375238 10.1016/j.phymed.2022.154524

[13] Malka D , Lièvre A , André T , Taïeb J , Ducreux M , Bibeau F . Immune scores in colorectal cancer: where are we? Eur J Cancer. 2020;140:105‐118. 33075623 10.1016/j.ejca.2020.08.024

[14] Mukherjee S , Joardar N , Sengupta S , Sinha Babu SP . Gut microbes as future therapeutics in treating inflammatory and infectious diseases: lessons from recent findings. J Nutr Biochem. 2018;61:111‐128. 30196243 10.1016/j.jnutbio.2018.07.010 PMC7126101

[15] Gagnière J . Gut microbiota imbalance and colorectal cancer. World J Gastroenterol. 2016;22:501‐518. 26811603 10.3748/wjg.v22.i2.501 PMC4716055

[16] Taghinezhad‐S S , Mohseni AH , Fu X . Intervention on gut microbiota may change the strategy for management of colorectal cancer. J Gastroenterol Hepatol. 2021;36:1508‐1517. 33295040 10.1111/jgh.15369

[17] Wang Y , Li H . Gut microbiota modulation: a tool for the management of colorectal cancer. J Transl Med. 2022;20:178. 35449107 10.1186/s12967-022-03378-8 PMC9022293

[18] Zhao H , He M , Zhang M , et al. Colorectal cancer, gut microbiota and traditional Chinese medicine: a systematic review. Am J Chin Med. 2021;49:805‐828. 33827382 10.1142/S0192415X21500385

[19] Rubinstein MR , Baik JE , Lagana SM , et al. *Fusobacterium nucleatum* promotes colorectal cancer by inducing wnt/beta‐catenin modulator annexin A1. EMBO Rep. 2019;20:e47638. 30833345 10.15252/embr.201847638 PMC6446206

[20] Chen S , Su T , Zhang Y , et al. *Fusobacterium nucleatum* promotes colorectal cancer metastasis by modulating KRT7‐AS/KRT7. Gut Microbes. 2020;11:511‐525. 31910722 10.1080/19490976.2019.1695494 PMC7524269

[21] Sakamoto Y , Mima K , Ishimoto T , et al. Relationship between *Fusobacterium nucleatum* and antitumor immunity in colorectal cancer liver metastasis. Cancer Sci. 2021;112:4470‐4477. 34464993 10.1111/cas.15126 PMC8586672

[22] Holt RA . Oncomicrobial vaccines: the potential for a *Fusobacterium nucleatum* vaccine to improve colorectal cancer outcomes. Cell Host Microbe. 2023;31:141‐145. 36634619 10.1016/j.chom.2022.11.014

[23] Padma S , Patra R , Sen Gupta PS , Panda SK , Rana MK , Mukherjee S . Cell surface fibroblast activation protein‐2 (Fap2) of *Fusobacterium nucleatum* as a vaccine candidate for therapeutic intervention of human colorectal cancer: an immunoinformatics approach. Vaccines. 2023;11:525. 36992108 10.3390/vaccines11030525 PMC10056511

[24] Sadowska A , Krętowski R , Szynaka B , Cechowska‐Pasko M , Car H . Metronidazole decreases viability of DLD‐1 colorectal cancer cell line. Cancer Biother Radiopharm. 2013;28:615‐622. 23777253 10.1089/cbr.2013.1485 PMC3777553

[25] Van Zuylen EM , Ferguson SA , Hughes A , Rennison D , Brimble MA , Cook GM . Disruption of metallostasis in the anaerobic human pathogen *Fusobacterium nucleatum* by the zinc ionophore PBT2. ACS Infect Dis. 2021;7:2285‐2298. 34259502 10.1021/acsinfecdis.0c00887

[26] Jiang SS , Xie YL , Xiao XY , et al. *Fusobacterium nucleatum*‐derived succinic acid induces tumor resistance to immunotherapy in colorectal cancer. Cell Host Microbe. 2023;31:781‐797. 37130518 10.1016/j.chom.2023.04.010

[27] Taniguchi G , Kajino K , Momose S , et al. The inhibitory effects of anti‐ERC/mesothelin antibody 22A31 on colorectal adenocarcinoma cells, within a mouse xenograft model. Cancers. 2022;14:2198. 35565327 10.3390/cancers14092198 PMC9101225

[28] Abed J , Maalouf N , Manson AL , et al. Colon cancer‐associated *Fusobacterium nucleatum* may originate from the oral cavity and reach colon tumors via the circulatory system. Front Cell Infect Microbiol. 2020;10:400. 32850497 10.3389/fcimb.2020.00400 PMC7426652

[29] Chang GR , Kuo CY , Tsai MY , et al. Anti‐cancer effects of zotarolimus combined with 5‐fluorouracil treatment in HCT‐116 colorectal cancer‐bearing BALB/c nude mice. Molecules. 2021;26:4683. 34361836 10.3390/molecules26154683 PMC8347948

[30] Li D , Xu X , Miao J , Cai J . MicroRNA‐125a inhibits tumorigenesis by targeting Smurf1 in colorectal carcinoma. FEBS Open Bio. 2019;9:1305‐1314. 10.1002/2211-5463.12680 PMC6609577 31141316

[31] Lee JG , Lee YR , Lee AR , Park CH , Han DS , Eun CS . Role of the global gut microbial community in the development of colitis‐associated cancer in a murine model. Biomed Pharmacother. 2021;135:111206. 33418307 10.1016/j.biopha.2020.111206

[32] Dapito DH , Mencin A , Gwak GY , et al. Promotion of hepatocellular carcinoma by the intestinal microbiota and TLR4. Cancer Cell. 2012;21:504‐516. 22516259 10.1016/j.ccr.2012.02.007 PMC3332000

[33] Liu D , Chen C , Cui M , Zhang H . miR‐140‐3p inhibits colorectal cancer progression and its liver metastasis by targeting BCL9 and BCL2. Cancer Med. 2021;10:3358‐3372. 33838016 10.1002/cam4.3840 PMC8124101

[34] Wang H , Wu X , Lezmi S , et al. Extract of *Ginkgo biloba* exacerbates liver metastasis in a mouse colon cancer xenograft model. BMC Complement Altern Med. 2017;17:516. 29197355 10.1186/s12906-017-2014-7 PMC5712166

[35] Kunzmann AT , Proença MA , Jordao HW , et al. *Fusobacterium nucleatum* tumor DNA levels are associated with survival in colorectal cancer patients. Eur J Clin Microbiol Infect Dis. 2019;38:1891‐1899. 31367996 10.1007/s10096-019-03649-1 PMC6778531

[36] Du G , Wang Y , Chen J , Deng Y , Han X , Tang G . Potential association between *Fusobacterium nucleatum* enrichment on oral mucosal surface and oral lichen planus. Oral Dis. 2020;26:122‐130. 31710746 10.1111/odi.13232

[37] Brenner H , Kloor M , Pox CP . Colorectal cancer. Lancet. 2014;383:1490‐1502. 24225001 10.1016/S0140-6736(13)61649-9

[38] Singh N , Baby D , Rajguru J , Patil P , Thakkannavar S , Pujari V . Inflammation and cancer. Ann Afr Med. 2019;18:121‐126. 31417011 10.4103/aam.aam_56_18 PMC6704802

[39] Armstrong E , Hemmerling A , Miller S , et al. Metronidazole treatment rapidly reduces genital inflammation through effects on bacterial vaginosis‐associated bacteria rather than lactobacilli. J Clin Invest. 2022;132(6):e152930. 35113809 10.1172/JCI152930 PMC8920324

[40] Martyniak A , Medyńska‐Przęczek A , Wędrychowicz A , Skoczeń S , Tomasik PJ . Prebiotics, probiotics, synbiotics, paraprobiotics and postbiotic compounds in IBD. Biomolecules. 2021;11:1903. 34944546 10.3390/biom11121903 PMC8699341

[41] Hashemi Goradel N , Heidarzadeh S , Jahangiri S , et al. *Fusobacterium nucleatum* and colorectal cancer: a mechanistic overview. J Cell Physiol. 2019;234:2337‐2344. 30191984 10.1002/jcp.27250

[42] Bullman S , Pedamallu CS , Sicinska E , et al. Analysis of fusobacterium persistence and antibiotic response in colorectal cancer. Science. 2017;358:1443‐1448. 29170280 10.1126/science.aal5240 PMC5823247

[43] Lin W , Zhu C , Yao J , Liu Y , Lin H , Liu Y . Multifactorial analysis of clinical prognosis of liver metastasis and vascular intervention combined with ablation in colorectal cancer. J Oncol. 2022;2022:1‐9. 10.1155/2022/9690401 PMC9206548 35726221

[44] Cui MY , Cui ZY , Zhao MQ , et al. The impact of helicobacter pylori infection and eradication therapy containing minocycline and metronidazole on intestinal microbiota. BMC Microbiol. 2022;22:321. 36581836 10.1186/s12866-022-02732-6 PMC9798553

[45] Wong SH , Yu J . Gut microbiota in colorectal cancer: mechanisms of action and clinical applications. Nat Rev Gastroenterol Hepatol. 2019;16:690‐704. 31554963 10.1038/s41575-019-0209-8

[46] Ibrahim A , Hugerth LW , Hases L , et al. Colitis‐induced colorectal cancer and intestinal epithelial estrogen receptor beta impact gut microbiota diversity. Int J Cancer. 2019;144:3086‐3098. 30515752 10.1002/ijc.32037 PMC6519213

[47] Toumazi D , Constantinou C . A fragile balance: the important role of the intestinal microbiota in the prevention and management of colorectal cancer. Oncology. 2020;98:593‐602. 32604093 10.1159/000507959

[48] Zhang Z , Cao H , Song N , Zhang L , Cao Y , Tai J . Long‐term hexavalent chromium exposure facilitates colorectal cancer in mice associated with changes in gut microbiota composition. Food Chem Toxicol. 2020;138:111237. 32145354 10.1016/j.fct.2020.111237

[49] Arnold CE , Isaiah A , Pilla R , et al. The cecal and fecal microbiomes and metabolomes of horses before and after metronidazole administration. PLoS One. 2020;15:e0232905. 32442163 10.1371/journal.pone.0232905 PMC7244109

[50] Nitzan O . Role of antibiotics for treatment of inflammatory bowel disease. World J Gastroenterol. 2016;22:1078‐1087. 26811648 10.3748/wjg.v22.i3.1078 PMC4716021

[51] Gotfred‐Rasmussen H , Stensvold CR , Ingham AC , et al. Impact of metronidazole treatment and *Dientamoeba Fragilis* colonization on gut microbiota diversity. J Pediatr Gastroenterol Nutr. 2021;73:23‐29. 33633081 10.1097/MPG.0000000000003096

[52] Dong J , Li Y , Xiao H , et al. Oral microbiota affects the efficacy and prognosis of radiotherapy for colorectal cancer in mouse models. Cell Rep. 2021;37:109886. 34706245 10.1016/j.celrep.2021.109886

[53] Hernández Ceruelos A , Romero‐Quezada LC , Ruvalcaba Ledezma JC , López Contreras L . Therapeutic uses of metronidazole and its side effects: an update. Eur Rev Med Pharmacol Sci. 2019;23:397‐401. 30657582 10.26355/eurrev_201901_16788

[54] Hassan M , Awadalla E , Ali R , Fouad S , Abdel‐Kahaar E . Thiamine deficiency and oxidative stress induced by prolonged metronidazole therapy can explain its side effects of neurotoxicity and infertility in experimental animals: effect of grapefruit co‐therapy. Hum Exp Toxicol. 2020;39:834‐847. 31997653 10.1177/0960327119867755

[55] Patra R , Das NC , Mukherjee S . Exploring the differential expression and prognostic significance of the COL11A1 gene in human colorectal carcinoma: an integrated bioinformatics approach. Front Genet. 2021;12:608313. 33597969 10.3389/fgene.2021.608313 PMC7882494

